# Isolation of Nematicidal Triterpenoid Saponins from *Pulsatilla koreana* Root and Their Activities against *Meloidogyne incognita*

**DOI:** 10.3390/molecules18055306

**Published:** 2013-05-08

**Authors:** Wei Li, Ya Nan Sun, Xi Tao Yan, Seo Young Yang, Suk Jun Lee, Hyo Jeung Byun, Chang Sup Moon, Byung Soo Han, Young Ho Kim

**Affiliations:** 1 College of Pharmacy, Chungnam National University, Daejeon 305-764, Korea; 2 Technical Research Institute, Dongbang Agro Co., LTD, Buyeo, Chungnam 323-932, Korea

**Keywords:** *Pulsatilla koreana*, triterpene saponin, *Meloidogyne incognita*, nematicidal activity

## Abstract

*Pulsatilla koreana*, a species endemic to Korea, is an important herb used in traditional medicine to treat amoebic dysentery and malaria. In the present study, 23 oleanane-type triterpenoid saponins **1**–**23** and eight lupane-type triterpenoid saponins **24**–**31** were isolated from the roots of *P. koreana.* Their structures were elucidated on the basis of spectroscopic data. The methanol extract and isolated compounds were next assessed for nematicidal activity against the root-knot nematode (*Meloidogyne incognita*). The methanol extract showed strong nematicidal activity after 48 h, with a LC_50_ value of 92.8 μg/mL. Compounds **2**, **5**, **9**, **20**, and **21** showed significant effects, with LC_50_ values ranging from 70.1 to 94.7 μg/mL after 48 h. These results suggest that triterpenoid saponins from *P. koreana* should be explored as potential natural nematicides for developing new agents to control root-knot nematode disease*.*

## 1. Introduction

Plant parasitic nematodes inflict serious damage on agricultural crops and plants. The primary pathogen, the root-knot nematode (*Meloidogyne* *incognita*), is known to predispose the host to attacks from soil borne fungal pathogens (secondary pathogens), resulting in a synergistic effect. In this phenomenon, *M. incognita*, by causing wounds in the roots of a plant through penetration of a stylet, provides a way for other fungal pathogens to enter and thus result in disease complex incidences [1,2,3]. In the past, synthetic compounds have mainly been used for plant protection, but many of these pesticides have side effects including residues in plants, contamination of groundwater, the potential for adverse ecological impacts from pesticide use, and the creation of a continuing need for the development of new nematode control strategies and products [4]. Recently, one alternative used has been to screen naturally occurring plant secondary compounds for appropriate nematicidal activity. Various nematicidal substances of plant origin such as triglycerides, sesquiterpenes, alkaloids, steroids, diterpenes, and flavonoids, have been identified in this way [5]. These compounds can be developed for use as nematicides themselves, or can serve as model compounds for the development of chemically synthesized derivatives with enhanced activity and reduced environmental impacts [6].

**Figure 1:**
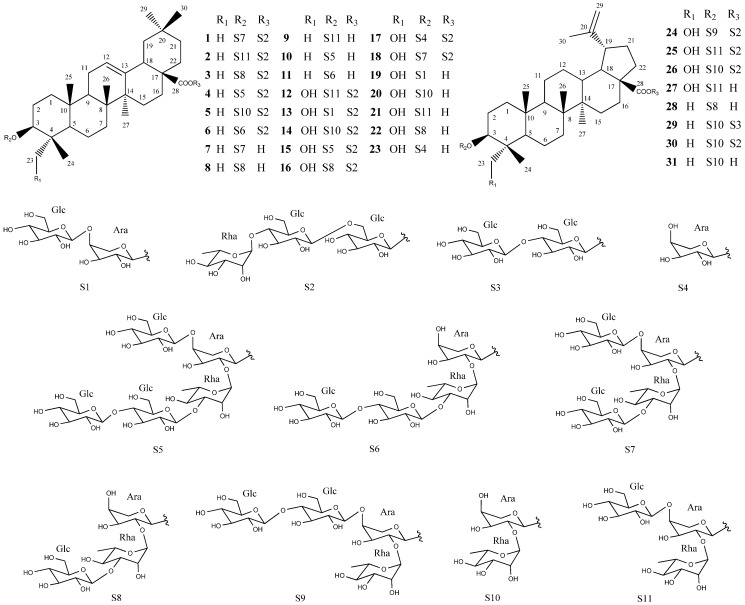
Structures of compounds **1**–**31** from the roots of *P. koreana*.

During our search for botanical pesticides from natural plants, we found that the methanol extract of *Pulsatilla koreana Nakai* (Ranuculaceae) has high nematicidal activity against *M. incognita*. *Pulsatilla koreana*, a hairy, tufted, perennial herb that grows in Korea, and is used as a traditional medicine to treat various maladies such as amoebic dysentery and malaria [7]. Several *Pulsatilla* species, including *P. ambigua*, *P. chinensis*, *P. dahurica* and *P. turczaninovii* have been employed to treat diarrhea, vaginal trichomoniasis, and bacterial infections. Pharmacological investigations have suggested that triterpene saponins are important bioactive components [8,9,10,11,12]. However, the composition of nematicidally active compounds in this plant has not been previously reported. In this study, 31 triterpenoid saponins were isolated (Figure 1) and their nematicidal activities against the root-knot nematode were evaluated.

## 2. Results and Discussion

### 2.1. Structure Elucidation of Compounds ***1**–**31***

In the present study, bioassay-guided isolation on the methanol extract of *P. koreana* roots was carried out to identify 31 triterpenoid saponins, which included 23 oleanane-type triterpenoid saponins **1**–**23** and eight lupane-type triterpenoid saponins **24**–**31***.* Their structures were elucidated as cernuoside A (**1**) [13], hederacholchiside E (**2**) [14], beesioside Q (**3**) [15], 3-*O*-*β*-d-glucopyranosyl (1→4)-*β*-d-glucopyranosyl (1→3)-*α*-l-rhamnopyranosyl (1→2)[*β*-d-glucopyranosyl(1→4)]-*α*-l-arabinopyranosyl oleanolic acid 28-*O*-*α*-l-rhamnopyranosyl (1→4)-*β*-d-glucopyranosyl (1→6)-*β*-d-glucopyranoside (**4**) [16], hederacoside B (**5**) [17], raddeanoside R17 (**6**) [18], 3-*O*-*β*-d-glucopyranosyl (1→3)-*α*-l-rhamno-pyranosyl (1→2) [*β*-d-glucopyranosyl (1→4)]-*α*-l-arabinopyranosyl oleanolic acid (**7**) [19], 3-*O*-*β*-d-glucopyranosyl (1→3)-*α*-l-rhamnopyranosyl (1→2)-*α*-l-arabinopyranosyl oleanolic acid (**8**) [20], raddeanoside R13 (**9**) [20], 3-*O*-*β*-d-glucopyranosyl (1→4)-*β*-d-glucopyranosyl (1→3)-*α*-l-rhamno-pyranosyl (1→2)[*β*-d-glucopyranosyl(1→4)]-*α*-l-arabinopyranosyl oleanolic acid (**10**) [21], 3-*O*-*β*-d-glucopyranosyl(1→4)-*β*-d-glucopyranosyl (1→3)-*α*-l-rhamnopyranosyl (1→2) [*β*-d-glucopyranosyl-(1→4)]-*α*-l-arabinopyranosyl oleanolic acid (**11**) [21], hederacholchiside F (**12**) [14], fatsiaside G (**13**) [22], pulsatilla saponin F (**14**) [23], pulsatilloside F (**15**) [24], patrinia saponin H3 (**16**) [25], hederasaponin D (**17**) [23], cernuoside B (**18**) [26], scabioside C (**19**) [20], hederoside C (**20**) [23], pulsatilla saponin D (**21**) [27], kalopanaxsaponin H (**22**) [28], scabioside A (**23**) [29], 23-hydroxy-3*β*-[(*O*-α-l-rhamnopyranosyl-(1→2)-*O*-[*β*-d-glucopyranosyl-(1→4)*-β*-d-glucopyranosyl-(1→4)]-*α*-l-arabino-pyranosyl)oxy]lup-20(29)-en-28-oic acid 28-*O*-*α*-l-rhamnopyranosyl-(1→4)-*O*-*β*-d-glucopyranosyl-(1→6)-*β*-d-glucopyranosyl ester (**24**) [30], pulsatilloside E (**25**) [20], anemoside B4 (**26**) [20], 23-hydroxy-3*β*-[(*O*-α-l-rhamnopyranosyl-(1→2)-*O*-[*β*-d-glucopyranosyl-(1→4)]-*α*-l-arabinopyranosyl)-oxy] lup-20(29)-en-28-oic acid (**27**) [12], 3*β*-[*O*-*β*-d-glucopyranosyl-(1→3)-*O*-α-l-rhamnopyranosyl-(1→2)-*O*-*α*-l-arabinopyranosyl)oxy] lup-20(29)-en-28-oic acid (**28**) [20], 3*β*-[(*O*-*α*-l-rhamno-pyranosyl-(1→2)-*α*-l-arabinopyranosyl)oxy] lup-20(29)-en-28-oic acid 28-*O*-*β*-d-glucopyranosyl-(1→6)-*β*-d-glucopyranosyl ester (**29**) [14], cussosaponin C (**30**) [30], betulinic acid 3*β*-*O*-*α*-l-rhamnopyranosyl-(1→2)-*O*-*α*-l-arabinopyranoside (**31**) [31] (Figure 1). Their structures were elucidated on the basis of spectroscopic data and comparison of 1D- and 2D-NMR and mass spectral data with reported values.

Among the isolated triterpenoid saponins, compounds **4**, **6**, **7**, **10**, **11**, **14**, **24**, and **31** were isolated from *P. koreana* for the first time. Compounds **1**–**6**, **10**–**12**, **16**, **18**, **24**–**26**, **30**, and **31** were only found previously in *P. chinensis* and *P. cernua* among in the genus *Pulsatilla*, suggesting a genetic relationship between them [11,13,16,21]. Additionally, compounds **27**–**29** have not been reported in other species of *Pulsatilla*, they will probably become a chemotaxonomic marker for *P. koreana*. This is the first integrated chemical investigation of triterpenoid saponins from *P. koreana* root.

### 2.2. Nematicidal Activity

**Figure 2:**
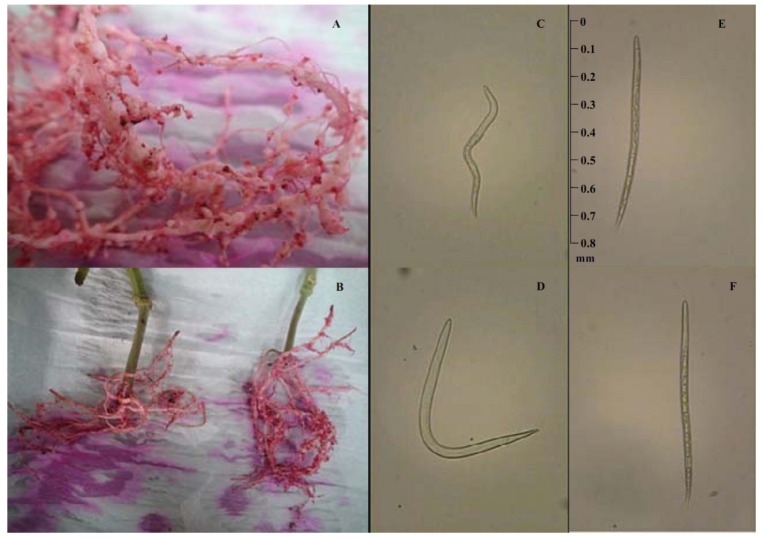
Muskmelon roots infested with *M. incognita* (**A** and **B**); Juveniles and adults of *M. incognita* treated with water and with compounds. A healthy, active nematode (**C** and **D**), compounds treated nematode with disrupted internal structures (**E** and **F**) and an almost empty somatocysts of the dead nematode after 72 h.

An investigation on the nematicidal activity of the methanol extract and isolated compounds of *P. koreana* root against *M. incognita* was conducted. The activity of all samples was also examined separately. The internal structures of the nematodes were disintegrated and many vacuoles formed within their bodies during the experiments. After 72 h, only residual, empty somatocysts of the dead nematodes could be observed (Figure 2E,F). The methanol extract of *P. koreana* showed strong nematicidal activity after 48 h, with a LC_50_ value of 92.8 μg/mL compared with positive control (72.7 μg/mL) (Table 1). Moreover, the nematicidal activity of methanol extract dramatically increased 24 h after the treatment. 

The isolated compounds were tested for *in vitro* nematicidal activity against *M. incognita*. Of these, compounds **9** and **20** exhibited strong activity after 24 h, with LC_50_ values of 88.7 and 92.4 μg/mL, respectively, compared with the positive control, fosthiazate (78.6 μg/mL). Compounds **2**, **5**, **12**, **14**, and **21** showed moderate effects, with LC_50_ values ranging from 103.9 to 186.7 μg/mL, and compounds **3**, **8**, **16**, and **22**–**31** exhibited weak activities (LC_50_ > 200 μg/mL). After 48 h, compounds **2**, **5**, **9**, **20**, and **21** showed significant effects, with LC_50_ values ranging from 70.1 to 94.7 μg/mL. Compounds **9** (2.1 g, 1.05%), **20** (120.0 mg, 0.06%), and **21** (196.0 mg, 0.10%), which are major triterpenoid saponin components, exhibited the same level of mortalities as fosthiazate (Table 1). After 72 h, the nematicidal activity effects of compounds **2**, **5**, **9**, **20**, and **21** showed no significant progress, suggesting that the isolated compounds exhibited activity within 48 h after treatment.

**Table 1:** Nematicidal activity of isolated compounds and methanol extract of *P. koreana* root against *Meloidogyne incognita*.

Compound	24 h	48 h	72 h
	LC_50_ (μg/mL) *	LC_50_ (μg/mL) *	LC_50_ (μg/mL) *
**2**	136.7 ± 38.6	94.7 ± 27.4	94.5 ± 25.6
**3**	>200	>200	>200
**5**	126.8 ± 29.7	88.9 ± 32.9	87.5 ± 19.8
**8**	>200	>200	169.2 ± 38.9
**9**	88.7 ± 21.9	75.8 ± 22.6	72.3 ± 24.4
**12**	177.0 ± 49.2	134.7 ± 47.6	131.7 ± 28.3
**14**	186.7 ± 56.4	127.5 ± 43.7	123.8 ± 36.7
**16**	>200	>200	>200
**20**	92.4 ± 14.6	70.1 ± 28.6	69.7 ± 18.8
**21**	103.9 ± 36.2	79.9 ± 18.3	77.6 ± 22.1
**22**	>200	>200	184.3 ± 36.7
**25**	>200	>200	>200
**26**	>200	>200	>200
**27**	>200	>200	>200
**28**	>200	>200	>200
**30**	>200	>200	>200
**31**	>200	>200	>200
MeOH ext.	106.7 ± 35.8	92.8 ± 27.6	86.5 ± 22.8
Fosthiazate **	78.6 ± 19.8	72.7 ± 21.6	71.4 ± 17.6

* All data represent the mean ± SD of at least three independent experiments performed in triplicates; ** Positive control.

A previous study reported that *P. koreana* showed significant activity against the root-knot nematode, but the active constituents were not determined [32]. To the best of our knowledge, this is the first report of nematicidal active constituents from *P. koreana*. In the structure-activity relationships of oleanane-type triterpenoid saponins **1**–**22**, compounds **9**, **20** and **21** showed the strongest nematicidal activity against *M. incognita*, and their structures were similar. When the sugar unit at C-3 of the aglycone was linked to a rhamnose-arabinose or rhamnose-(glucose-)arabinose chain, the nematicidal activity increased significantly when compared to other sugar chains. Although compounds **2**, **5**, **12**, and **14** also link a rhamnose-arabinose or rhamnose-(glucose-)arabinose chain, their effects were weaker than those of compounds **9**, **20** and **21**, suggesting that a carboxyl group at position C-28 of aglycone is a key functional element. This suggestion was supported by comparing with previous work [9,33,34]. These data may be useful to evaluate the structure-activity relationships of other triterpenoid saponins, and to develop nematicidal activity against *M. incognita*.

## 3. Experimental

### 3.1. General Procedures

Optical rotations were determined using a Jasco DIP-370 automatic polarimeter. The FT-IR spectra were measured using a Jasco Report-100 infrared spectrometer. The NMR spectra were recorded using a JEOL ECA 600 spectrometer (^1^H, 600 MHz; ^13^C, 150 MHz), and ESI-MS spectra were obtained using a JEOL JMS-T100LC spectrometer. Column chromatography was performed using a silica gel (Kieselgel 60, 70–230, and 230–400 mesh, Merck, Darmstadt, Germany) and YMC RP-18 resins, and thin layer chromatography (TLC) was performed using pre-coated silica-gel 60 F_254_ and RP-18 F_254_S plates (both 0.25 mm, Merck).

### 3.2. Plant Material

Dried roots of *P. koreana* were purchased from herbal market, Kumsan, Chungnam, Korea in March 2009 and identified by one of the authors (Prof. Young Ho Kim). A voucher specimen (CNU 09106) was deposited at the Herbarium of College of Pharmacy, Chungnam National University, Daejeon, Korea.

### 3.3. Extraction and Isolation

Dried roots of *P. koreana* (2.0 kg) were extracted with MeOH under reflux for 10 h (7 L × 3 times) to yield 500.0 g of extract. This extract was suspended in water and partitioned with ethyl acetate to yield the corresponding ethyl acetate (37.0 g) and water (463.0 g) extracts. The water extract was partitioned with *n*-BuOH to yield the *n*-BuOH (130.0 g) extract. The ethyl acetate extract was subjected to silica gel column chromatography with a gradient of CHCl_3_/MeOH (50:1, 20:1,10: 1 and 1:1; 2 L for each step) to give 6 fractions (Fr. E1–E6). The fraction E4 was separated using an YMC column with a MeOH/actone/H_2_O (0.25:0.3:1–1.3:1.3:1, 1.2 L for each step) elution solvent to give compounds **23** (75.0 mg) and **31** (62.0 mg). The fraction E5 was separated using an YMC column with a MeOH/H_2_O (3.2:1, 1.4 L) elution solvent to give compound **11** (19.0 mg). The fraction E6 was separated using an YMC column with a MeOH/H_2_O (2.7:1, 1.5 L) elution solvent to give compound **27** (28.0 mg). 

The *n*-BuOH extract was subjected to silica gel column chromatography with a gradient of CHCl_3_/MeOH/H_2_O (5:1:0.1, 2:1:0.1 and 0:1:0; 3 L for each step) to give 6 fractions (Fr. B1–B6). The fraction B3 was separated using a silica gel column with CHCl_3_/MeOH/H_2_O (5:1:0.1, 4:1:0.1 and 3:1:0.1, 1 L for each step) to give four sub-fractions (Fr. B3.1–B3.4). Fraction B3.1 was separated using an YMC column with a MeOH/H_2_O (4.5:1, 1.1 L) elution solvent to give compound **20** (120.0 mg). Fraction B3.2 was separated using an YMC column with a MeOH/acetone/H_2_O (2.5:0.7:1, 2.5 L) elution solvent to give compounds **8** (78.0 mg), **19** (30.0 mg) and **28** (12.0 mg). Fraction B3.3 was separated using an YMC column with a MeOH/acetone/H_2_O (1.5:0.7:1, 750 mL) elution solvent to give compound **22** (180.0 mg). Fraction B3.4 was separated using an YMC column with a MeOH/acetone/H_2_O (2:0.5:1, 2.5 L) elution solvent to give compounds **9** (2.1 g) and **25** (12.0 mg). The fraction B4 was separated using a silica gel column with CHCl_3_/MeOH/H_2_O (3.5:1:0.1, 2:1:0.1 and 1:1:0.2, 1 L for each step) to give four sub-fractions (Fr. B4.1–B4.4). Fraction B4.2 was further chromatographed on RP chromatography column with acetone/MeOH/H_2_O (0.5:1:1.8, 1.5 L) to yield compound **29** (130.0 mg). Fraction B4.3 was separated using an YMC column with a MeOH/acetone/H_2_O (10.5:1:1–0.85:2:1, each 550 mL) elution solvent to give compounds **10** (25.0 mg), **26** (7.0 mg) and **30** (20.0 mg).

The water extract was chromatographed on a column of highly porous polymer (Diaion HP-20) and eluted with H_2_O and MeOH, successively, to give four fractions (Fr. W1–W4). Fraction W3 was subjected to silica gel column chromatography with a gradient of CHCl_3_/MeOH/H_2_O (6:1:0.1, 4:1:0.1, 2:1:0.1 and 0:1:0; 4 L for each step) to give six fractions (Fr. W3.1–W3.6). Fraction W3.3 using an YMC column with a MeOH/acetone/H_2_O (1:0.3:1–1:0.4:1.4, 650 mL for each step) elution solvent to give compounds **2** (50.0 mg), **3** (44.0 mg), **13** (17.0 mg), **14** (77.0 mg), and **16** (38.0 mg). Fraction W3.4 was separated using an YMC column with a MeOH/H_2_O (1.3:1–2.5:1, 750 mL for each step) elution solvent to give compounds **1** (44.0 mg) and **12** (110.0 mg). Fraction W3.5 was separated using an YMC column with an acetone/MeOH/H_2_O (0.25:1:1–0.32:1:1, 600 mL for each step) elution solvent to give compounds **4** (78.0 mg) and **18** (460.0 mg). Fraction W3.6 was separated using a silica gel column with CHCl_3_/MeOH/H_2_O (1.2:1:0.15, 1.5 L) to give compounds **15** (130.0 mg) and **24** (4.0 mg). Fraction W4 was subjected to silica gel column chromatography with a gradient of CHCl_3_/MeOH/H_2_O (2.5:1:0.1, 1.5:1:0.15 and 0:1:0; 3 L for each step) to give 3 fractions (Fr. W4.1–W4.3). Fraction W4.1 was further chromatographed on RP chromatography column with acetone/MeOH/H_2_O (0.7:1.5:1–1:2:1, 1 L for each step) to yield compounds **7** (12.0 mg) and **21** (196.0 mg). Fraction W4.2 was further chromatographed on RP chromatography column with acetone-MeOH-H_2_O (0.6:1:1–1:1.7:1, 750 mL for each step) to yield compounds **5** (300.0 mg) and **17** (12.0 mg). Compound **6** (36.0 mg) was isolated from W4.3 using RP chromatography column with acetone/MeOH/H_2_O (0.3:1.7:1). 

*Raddeanoside R13* (**9**): White powder; ESI-MS *m/z* 895 [M−H]^−^; ^1^H-NMR of aglycone (600 MHz, pyridine-*d_5_*) *δ*: 1.05 (1H, m, H-1a), 1.52 (1H, m, H-1b), 2.00 (1H, m, H-2a), 2.23 (1H, m, H-2b), 4.29 (1H, dd, *J =* 3.6, 11.0 Hz, H-3), 0.72 (1H, d, *J =* 12.0 Hz, H-5), 1.60 (2H, m, H-6), 1.29 (1H, m, H-7a), 1.40 (1H, m, H-7b), 1.61 (1H, m, H-9), 0.88 (2H, m, H-11), 5.35 (1H, br s, H-12), 1.16 (1H, m, H-15a), 2.27 (1H, m, H-15b), 1.90 (1H, m, H-16a), 2.05 (1H, m, H-16b), 3.31 (1H, d, *J =* 12.0 Hz, H-18), 1.21 (1H, m, H-19a), 1.67 (1H, m, H-19b), 0.90 (1H, m, H-21a), 1.09 (1H, m, H-21b), 1.75 (1H, m, H-22a), 1.85 (1H, m, H-22b), 1.26 (3H, s, H-23), 1.12 (3H, s, H-24), 0.84 (3H, s, H-25), 1.00 (3H, s, H-26), 1.32 (3H, s, H-27), 0.97 (3H, s, H-29), 0.85 (3H, s, H-30); ^13^C-NMR of aglycone (150 MHz, pyridine-*d_5_*) *δ*: 39.2 (C-1), 27.0 (C-2), 89.1 (C-3), 39.9 (C-4), 56.3 (C-5), 18.7 (C-6), 33.5 (C-7), 40.1 (C-8), 48.4 (C-9), 37.4 (C-10), 24.0 (C-11), 123.0 (C-12), 145.3 (C-13), 42.5 (C-14), 28.6 (C-15), 24.0 (C-16), 47.0 (C-17), 42.3 (C-18), 46.8 (C-19), 31.3 (C-20), 34.6 (C-21), 33.5 (C-22), 28.5 (C-23), 17.5 (C-24), 15.9 (C-25), 17.7 (C-26), 26.5 (C-27), 180.8 (C-28), 33.6 (C-29), 24.1 (C-30); ^1^H-NMR of sugar moieties (600 MHz, pyridine-*d_5_*) *δ*: 4.85 (1H, d, *J* = 6.5 Hz, Ara-1), 4.15 (1H, m, Ara-2), 4.35 (1H, m, Ara-3), 4.24 (1H, m, Ara-4), 3.96 (1H, m, Ara-5a), 4.40 (1H, m, Ara-5b), 6.23 (1H, br s, Rha-1), 4.90 (1H, m, Rha-2), 4.74 (1H, m, Rha-3), 4.50 (1H, m, Rha-4), 4.95 (1H, m, Rha-5), 1.57 (1H, d, *J =* 6.0 Hz, Rha-6), 5.48 (1H, d, *J* = 7.7 Hz, Glc-1), 3.92 (1H, m, Glc-2), 4.28 (1H, m, Glc-3), 4.31 (1H, m, Glc-4), 4.08 (1H, m, Glc-5), 3.82 (1H, m, Glc-6a), 4.44 (1H, m, Glc-6b); ^13^C-NMR of sugar moieties (150 MHz, pyridine-*d_5_*) *δ*: 105.9 (Ara-1), 76.7 (Ara-2), 74.5 (Ara-3), 80.2 (Ara-4), 65.0 (Ara-5), 102.1 (Rha-1), 72.0 (Rha-2), 72.9 (Rha-3), 74.5 (Rha-4), 70.2 (Rha-5), 18.8 (Rha-6), 106.9 (Glc-1), 75.1 (Glc-2), 78.9 (Glc-3), 71.6 (Glc-4), 79.2 (Glc-5), 62.9 (Glc-6).

*Hederoside C* (**20**): White powder; ESI-MS *m/z* 749 [M−H]^−^; ^1^H-NMR of aglycone (600 MHz, pyridine-*d_5_*) *δ*: 1.05 (1H, m, H-1a), 1.52 (1H, m, H-1b), 2.05 (1H, m, H-2a), 2.44 (1H, m, H-2b), 4.35 (1H, dd, *J =* 3.6, 11.0 Hz, H-3), 1.75 (1H, d, *J =* 12.0 Hz, H-5), 1.53 (2H, m, H-6), 1.26 (1H, m, H-7a), 1.55 (1H, m, H-7b), 1.77 (1H, m, H-9), 1.96 (2H, m, H-11), 5.50 (1H, br s, H-12), 1.15 (1H, m, H-15a), 2.27 (1H, m, H-15b), 1.90 (1H, m, H-16a), 2.09 (1H, m, H-16b), 3.28 (1H, d, *J =* 12.0 Hz, H-18), 1.20 (1H, m, H-19a), 1.69 (1H, m, H-19b), 0.91 (1H, m, H-21a), 1.08 (1H, m, H-21b), 1.73 (1H, m, H-22a), 1.87 (1H, m, H-22b), 4.02 (1H, d, *J =* 12.0 Hz, H-23a), 4.50 (1H, d, *J =* 12.0 Hz, H-23b), 1.04 (3H, s, H-24), 0.96 (3H, s, H-25), 1.01 (3H, s, H-26), 1.25 (3H, s, H-27), 0.93 (3H, s, H-29), 0.94 (3H, s, H-30); ^13^C-NMR of aglycone (150 MHz, pyridine-*d_5_*) *δ*: 39.3 (C-1), 26.6 (C-2), 81.4 (C-3), 43.8 (C-4), 48.5 (C-5), 18.5 (C-6), 33.6 (C-7), 40.1 (C-8), 48.1 (C-9), 37.2 (C-10), 24.2 (C-11), 123.0 (C-12), 145.3 (C-13), 42.5 (C-14), 28.7 (C-15), 24.0 (C-16), 47.0 (C-17), 42.3 (C-18), 46.8 (C-19), 31.3 (C-20), 34.5 (C-21), 33.2 (C-22), 64.3 (C-23), 14.3 (C-24), 16.4 (C-25), 17.8 (C-26), 26.5 (C-27), 180.8 (C-28), 33.6 (C-29), 24.1 (C-30); ^1^H-NMR of sugar moieties (600 MHz, pyridine-*d_5_*) *δ*: 5.22 (1H, d, *J* = 6.5 Hz, Ara-1), 4.25 (1H, m, Ara-2), 4.35 (1H, m, Ara-3), 4.34 (1H, m, Ara-4), 4.00 (1H, m, Ara-5a), 4.40 (1H, m, Ara-5b), 6.29 (1H, br s, Rha-1), 4.72 (1H, m, Rha-2), 4.55 (1H, m, Rha-3), 4.28 (1H, m, Rha-4), 4.37 (1H, m, Rha-5), 1.56 (1H, d, *J =* 6.0 Hz, Rha-6); ^13^C-NMR of sugar moieties (150 MHz, pyridine-*d_5_*) *δ*: 104.9 (Ara-1), 76.2 (Ara-2), 75.2 (Ara-3), 69.8 (Ara-4), 66.2 (Ara-5), 102.1 (Rha-1), 72.8 (Rha-2), 72.9 (Rha-3), 74.5 (Rha-4), 70.1 (Rha-5), 18.9 (Rha-6).

*Pulsatilla saponin* D (**21**): White powder; ESI-MS *m/z* 911 [M−H]^−^; ^1^H-NMR of aglycone (600 MHz, pyridine-*d_5_*) *δ*: 1.07 (1H, m, H-1a), 1.54 (1H, m, H-1b), 2.05 (1H, m, H-2a), 2.40 (1H, m, H-2b), 4.38 (1H, dd, *J =* 3.6, 11.0 Hz, H-3), 1.75 (1H, d, *J =* 12.0 Hz, H-5), 1.53 (2H, m, H-6), 1.26 (1H, m, H-7a), 1.55 (1H, m, H-7b), 1.77 (1H, m, H-9), 1.96 (2H, m, H-11), 5.50 (1H, br s, H-12), 1.15 (1H, m, H-15a), 2.27 (1H, m, H-15b), 1.90 (1H, m, H-16a), 2.09 (1H, m, H-16b), 3.30 (1H, d, *J =* 12.0 Hz, H-18), 1.20 (1H, m, H-19a), 1.69 (1H, m, H-19b), 0.90 (1H, m, H-21a), 1.08 (1H, m, H-21b), 1.73 (1H, m, H-22a), 1.87 (1H, m, H-22b), 4.05 (1H, d, *J =* 12.0 Hz, H-23a), 4.52 (1H, d, *J =* 12.0 Hz, H-23b), 1.04 (3H, s, H-24), 0.96 (3H, s, H-25), 1.01 (3H, s, H-26), 1.25 (3H, s, H-27), 0.93 (3H, s, H-29), 0.94 (3H, s, H-30); ^13^C-NMR of aglycone (150 MHz, pyridine-*d_5_*) *δ*: 39.1 (C-1), 26.4 (C-2), 82.4 (C-3), 43.8 (C-4), 47.9 (C-5), 18.5 (C-6), 33.6 (C-7), 40.1 (C-8), 47.9 (C-9), 37.3 (C-10), 24.2 (C-11), 123.0 (C-12), 145.3 (C-13), 42.5 (C-14), 28.7 (C-15), 24.0 (C-16), 47.0 (C-17), 42.3 (C-18), 46.8 (C-19), 31.3 (C-20), 34.5 (C-21), 33.2 (C-22), 64.8 (C-23), 13.9 (C-24), 16.4 (C-25), 17.8 (C-26), 26.5 (C-27), 180.8 (C-28), 33.6 (C-29), 24.1 (C-30); ^1^H-NMR of sugar moieties (600 MHz, pyridine-*d_5_*) *δ*: 4.93 (1H, d, *J* = 6.5 Hz, Ara-1), 4.15 (1H, m, Ara-2), 4.35 (1H, m, Ara-3), 4.24 (1H, m, Ara-4), 3.96 (1H, m, Ara-5a), 4.40 (1H, m, Ara-5b), 5.82 (1H, br s, Rha-1), 4.72 (1H, m, Rha-2), 4.55 (1H, m, Rha-3), 4.28 (1H, m, Rha-4), 4.37 (1H, m, Rha-5), 1.64 (1H, d, *J =* 6.0 Hz, Rha-6), 5.26 (1H, d, *J* = 7.0 Hz, Glc-1), 3.95 (1H, m, Glc-2), 4.20 (1H, m, Glc-3), 4.89 (1H, m, Glc-4), 4.10 (1H, m, Glc-5), 4.27 (1H, m, Glc-6a), 4.52 (1H, m, Glc-6b); ^13^C-NMR of sugar moieties (150 MHz, pyridine-*d_5_*) *δ*: 104.2 (Ara-1), 76.0 (Ara-2), 74.9 (Ara-3), 80.3 (Ara-4), 65.3 (Ara-5), 101.5 (Rha-1), 72.1 (Rha-2), 72.3 (Rha-3), 73.9 (Rha-4), 69.5 (Rha-5), 18.4 (Rha-6), 106.6 (Glc-1), 75.3 (Glc-2), 78.3 (Glc-3), 71.0 (Glc-4), 78.6 (Glc-5), 62.2 (Glc-6).

### 3.4. Nematicidal Assay

A soil sample was collected from a pure culture of *M. incognita* and maintained on muskmelon roots in a greenhouse in Gimcheon (Gyeongbuk, Korea). Emerged larvae were collected from the soil using the Baermann funnel technique. Larvae were placed in a cavity block with water for a bioassay after they had been counted in a counting chamber. MeOH extract and the compounds isolated from *P. koreana* (5.0 mg) were prepared in 0.1 mL dimethyl sulfoxide (DMSO) and then diluted in water to obtain various concentration preparations (50–200 μg/mL). The standard nematicide fosthiazate was used for the comparison [35]. As a negative control of nematicidal activity, 5% DMSO was used. Approximately 40–60 freshly hatched second-stage juveniles in 450 μL of water and 50 μL of each of the 12 compounds at different concentrations were introduced into 24-well plates with three replicates performed. Plates were kept at room temperature (23–25 °C) under laboratory conditions. Inactive nematodes (dead nematodes) counted after 24, 48, and 72 h [36]. After the last count, inactive juveniles were maintained in distilled water for 72 h to observe their revival (Figure 2). Five repetitions for each treatment were performed using water as a control. 

## 4. Conclusions

In present study, bioassay-guided chromatographic fractionation and isolation were successfully used to yield 31 triterpenoid saponins from *P. koreana* roots. Three of the isolated compounds, raddeanoside R13 (**9**), hederoside C (**20**), and pulsatilla saponin D (**21**) may have potential as natural nematicides or as lead molecules for developing new nematicides to control root-knot nematode disease caused by *M. incognita.*
